# Racial Disparities in Substance Use Treatment Completion Among Older Adults

**DOI:** 10.1093/geroni/igaa051

**Published:** 2020-10-26

**Authors:** Zainab D Suntai, Lewis H Lee, James D Leeper

**Affiliations:** 1 School of Social Work, University of Alabama, Tuscaloosa, USA; 2 College of Community Health Sciences, University of Alabama, Tuscaloosa, USA

**Keywords:** Alcohol use disorder, Black, Hispanic, Treatment adherence

## Abstract

**Background and Objectives:**

Racial disparities in substance use among young adults have been well documented in the substance use literature, but little attention has been paid to older adults. While being an older adult is positively associated with substance use treatment completion, racial disparities in treatment completion have yet to be examined. The purpose of this study was to determine to what extent racial disparities exist in substance use treatment completion among older adults (65 and older).

**Research Design and Methods:**

This cross-sectional study utilized data from the most recent Treatment Episode Data from the Substance Abuse and Mental Health Services Administration, which documents discharges from a publicly funded substance use treatment program in the United States. A total of 17,942 older adults reported to a substance use treatment program in 2017 and 6,653 met the criteria for the study. Chi-squared tests were used to analyze group differences and a binary logistic regression was used to predict substance use treatment completion.

**Results:**

Results show that Black older adults were 37% less likely to complete a substance use treatment program than Whites (OR = 0.630) while Hispanic older adults were 26% more likely to complete a substance use treatment program than Whites (OR = 1.26).

**Discussion and Implications:**

These results support the findings from similar studies with younger adults and support the theory that racial disparities are prevalent across the life span. Although Hispanics had a higher treatment completion rate than Whites, this is likely a reflection of *familismo*, where decisions about health treatments is a group process and a steady network of family members are available to provide advice and encouragement. The significant disparity observed between Black and White older adults suggest a need to consider cultural, historical, and systemic factors that affect voluntary termination of substance use treatment among Black older adults.

**Translational Significance:** The results of this study indicate a significant disparity in substance use treatment completion between White and Black older adults, which has also been observed among younger adults. In practice, this suggests a need to conduct psychosocial assessments at treatment entry to determine barriers to completion specific to older adults who are minorities and attempt to address those barriers in conjunction with substance use treatment to ensure completion.

Substance use is a burgeoning public health issue among older adults. Due to the variation in symptomology among this population compared to younger adults, substance use disorders among older adults have been termed an invisible epidemic (Alpert, 2014). In spite of the fact that substance use research has primarily targeted young adults, older adults are an overlooked group that are at great risk of substance use dependency due to the multitude of afflictions that come with aging (Briggs et al., 2011). While substance use has been found to decrease with age (Mattson et al., 2017), factors such as pain associated with chronic illnesses, social isolation, depression, and other psychosocial issues that older adults face create a need for coping mechanisms that often involve drugs and alcohol (Heron, 2019; Reid et al., 2015). With the aging baby boomer cohort’s increasing exposure to and usage of drugs (Mattson et al., 2017), the number of older adults in need of substance use treatment is on the rise.

Between 2002 and 2012, the proportion of older adults admitted to a substance use treatment program increased from 3.4% to 7.0% (Chhatre et al., 2017). In addition to the increase in proportion, the usage of illicit substances such as cocaine and heroin also increased during that time period, though alcohol remained the primary substance of use among older adults (Chhatre et al., 2017). Gfroerer and colleagues (2003) predicted that the number of older adults who will need substance use treatment will see an upsurge from 1.7 million in 2001 to 4.4 million in 2020. While these projections of substance use among older adults are evident, studies assessing racial differences in the prevalence of substance use and treatment completion among older adults is limited.

In the general population, significant racial disparities in substance use prevalence and treatment completion have been noted. Minorities have been found to have higher cases of substance use and are also less likely to complete a substance use treatment program (Mennis & Stahler, 2016). Among adolescents, Black and Hispanic teenagers reported having less specialty and informal care for their substance use disorder than their White peers (Alegria et al., 2011) and in studies comparing the likelihood of completing an alcohol treatment program, African Americans and Hispanics were found to be significantly less likely to complete than Whites (Bluthenthal et al., 2007).

These disparities are also marked by systemic levels of racial injustice that moderate the prevalence of substance use disorders among minorities and the lower treatment completion rates. Minorities represent a staggering percentage of the homeless population in the United States and are influenced by racist redlining policies that contribute to housing inequity to date (Rutan & Glass, 2018). Minorities have also been found to have higher rates of chronic illnesses, lower educational attainment, and have almost twice the proportion of households below the poverty line compared to Whites (Cunningham et al., 2017; Price et al., 2013). These systemic issues of social justice undoubtedly influence the outcomes of addiction treatment as higher socioeconomic status is a protective factor for treatment completion. For example, one study found that African Americans had a 17.5% substance use treatment completion rate while Whites had a 26.7% completion rate, and this disparity was attributed to differences in socioeconomic factors such as homelessness, employment, and health insurance (Jacobson et al., 2007). Black/African American adults have also reported delays in substance use treatment entry as compared to Whites in a study assessing racial and socioeconomic disparities in substance use treatment (Lewis et al., 2018).

Other systemic disparities also exist in terms of health insurance and geographic access to a substance use treatment facility. A study by Cummings and colleagues found that only 60% of U.S. counties have an outpatient substance abuse treatment facility that accepts Medicaid and counties with a higher percentage of black, rural, and underinsured residents are less likely to have a substance use treatment facility (Cummings et al., 2014). These geographic inequities particularly alienate minorities who are more likely to use Medicaid (Kaiser Family Foundation, 2019).

Although these disparities have been noted among younger adults, studies addressing differences among older adults are scarce. While there are few studies targeting older adults specifically, Vasilenko and colleagues (2017) have found higher prevalence of alcohol use disorder and tobacco use disorder among Black older adults compared to Whites and Hispanics, while Gurnack and Johnson (2002) found higher usage of illicit drugs among African American older adults. Chhatre and colleagues have also found that between 2002 and 2012, the percentage of African American older adults admitted to substance use treatment programs increased from 21% to 28% while the proportion of non-Hispanic Whites decreased by 3% (2017). While these studies have found notable differences in the prevalence of substance use among older adults, there are no studies that examine racial disparities substance use treatment completion rates among older adults. The increasing trend in older adults seeking substance use treatment and the racial disparities found in younger adults posits a need to evaluate the extent of these differences across the life span. The purpose of this study is to address this gap in literature and examine racial differences in substance use treatment completion among older adults by comparing treatment completers with those who voluntarily terminate treatment. For the purpose of this study, treatment completion refers to the successful conclusion of a treatment plan as determined by the substance use treatment facility (SAMHSA, 2019b).

## Conceptual Framework

A number of factors have been proposed to explain the racial disparities in substance use treatment utilization and completion. These include socioeconomic status, criminal history, co-occurring mental health conditions, geographic availability of substance use treatment programs, and so forth (Choi et al., 2014; Cummings et al., 2014; Maremmani et al., 2017). To conceptualize the existence of other explanatory variables within this study, Andersen’s Healthcare Utilization Model provides a comprehensive guide that includes the factors that have been proposed in previous studies. The Andersen model was developed in the late 1960s by Ronald M. Andersen and has been widely used to address health disparities among underserved populations (Bonomi et al., 2009; Lee et al., 2017). The Andersen model suggests that a person’s use of health services is attributed to their predisposition to use services, enabling or impeding factors and the need for the health care service (Andersen, 1995). Predisposing factors include demographic measures such as age and gender, enabling or impeding factors include social capital such as family and community, and need factors include individual perception of health or official medical diagnosis (Andersen, 1995).

## Objectives and Research Question

Although substance use among older adults is still understudied, a number of studies have found disproportionately higher rates of substance use prevalence among minorities. While most studies have focused on identifying the rates of use, no studies have been identified that address the utilization and completion of substance use treatment programs among this population. Furthermore, racial differences in completion rates have been neglected, and research with younger adults suggests that there may be significant differences in the completion rates between racial groups. In this context, the purpose of this study is to fill the gap in literature by answering the following research question: To what extent do racial disparities exist in substance use treatment completion among older adults? In line with the research question, this study aims to determine if previously identified racial disparities in substance use treatment completion are consistent through older adulthood. We hypothesize that there will be a difference in the treatment completion rates between minorities and Whites.

## Method

### Data Source

Data on discharges from publicly funded substance use treatment programs were derived from the most recently available Treatment Episode Data Set—Discharges (TEDS-D) from the Substance Abuse and Mental Health Services Administration (SAMHSA), a national collection of annual discharges from substance use treatment programs in 2017. Data include demographic and substance use characteristics of individuals age 12 and older in substance use treatment facilities that report to state administrative data systems (SAMHSA, 2019b). TEDS-D only includes admissions to facilities that are licensed or certified by state agencies to provide substance use treatment services. Facilities reporting to TEDS-D are mostly those funded by state or drug agency funds, so the TEDS-D does not represent all substance use treatment facilities in the United States but is considered a nationally representative sample (SAMHSA, 2019b). The types of treatment programs in the data set include certified opioid treatment programs, community-based correctional programs, hospitals/Veterans Affairs hospitals/state hospitals, state-licensed/certified solo practitioners, state/correctional DUI/DWI providers, state divisional service centers, and private facilities (SAMHSA, 2019a).

Because this study is focused on racial disparities among older adults, only discharges of individuals aged 65 and older were included (*N* = 6,653). Older adult age range has been defined in a number of ways across the gerontology literature, but 65 and older was chosen as defined by the American Psychological Association (American Psychological Association, n.d.).

### Procedures

Each individual state is responsible for the aggregation of discharge data in any given year. Combined data from all agencies are then converted to meet TEDS standards by modifying the state data crosswalk. Once data are validated, all state reports are combined in the national TEDS database and available for data analysis (SAMHSA, 2019a).

### Variables

All variables used for the study were coded as one (1) to represent the presence of a characteristic and zero (0) to represent the absence of the characteristic. The code zero (0) represents the reference category and any number above zero are comparison categories.

#### Dependent variable

The main outcome variable of substance use treatment completion was coded as 1 for treatment completed and 0 for treatment voluntarily terminated. Other reasons for discharge included “terminated by facility,” “transferred to another facility,” “incarcerated,” “death,” and “other” but were excluded because these other options do not reflect a necessarily voluntary termination of substance use treatment by the individual. The purpose of this study is to determine racial differences in individuals who are completing the program or voluntarily terminating so these other options were not included as they could skew the causal mechanism of treatment completion as noted in a similar study by Mennis and Stahler (2016). It is important to note that “voluntary” termination of treatment may also reflect a number of external factors such as a need to return to employment, a family emergency, or financial/insurance reasons.

#### Independent variables

Guided by the Andersen model of health care utilization, explanatory variables to represent predisposing factors, enabling or impeding factors, and need factors were chosen. The main independent variable of race was coded to represent mutually exclusive and exhaustive categories. Non-Hispanic Black/African Americans were coded as Black, non-Hispanic Whites were coded as White, and all older adults who identified as Hispanic were coded as Hispanic, regardless of race or ethnicity. Blacks/African Americans were chosen because they have been identified as having the greatest disparity compared to Whites in other studies with younger adults, and Hispanics were included even though other studies did not find practically significant differences in treatment completion compared to Whites (Acevedo et al., 2015; Archibald, 2007; Mennis & Stahler, 2016; Saloner & Cook, 2013). The three included races represent the top three racial groups in the United States (Kaiser Family Foundation, 2020). Other races were not included because they represented a very small percent of substance use treatment admissions for older adults in 2017. Hereafter, non-Hispanic Blacks will be referred to as Black/African American and non-Hispanic Whites will be referred to as Whites. All Hispanics regardless of race or ethnicity will be referred to as Hispanics.

Because participants were only described as 65 and older, age could not be used as a part of the analysis as a predisposing factor so gender was selected as a predisposing factor along with race and because females have been found to have better treatment outcomes than males (Marsh et al., 2004). Gender was coded as 1 = male and 0 = female. Marital status, education, and employment were chosen as enabling factors because being married, having higher educational level, and having employment have been identified as protective factors for substance use (Heinz et al., 2009; Mutter et al., 2015). Marital status was coded as 0 = married, 1 = not married, and 2 = separated/divorced/widowed. Education was coded as 0 = has postsecondary education, 1 = completed high school, and 2 = did not complete high school. Employment was coded as 0 = employed, 1 = unemployed, and 2 = not in labor force. Participants that reported either having full-time or part-time employment were considered employed. Those who reported “not in labor force” were either retired, a student, disabled, a homemaker, or a resident of an institution such as hospitals, jails or prisons (SAMHSA, 2019b). These reasons for “not in labor force” were not examined as variables in the analysis.

The need factors chosen were the type of primary substance used and frequency of use, both of which could influence perceived need for substance use treatment. Those with alcohol as their primary substance have been found to have higher treatment completion rates compared to illicit drug users and frequency of use affects substance use treatment outcomes (Mennis & Stahler, 2016; Proctor & Herschman, 2014). Primary substance was coded as 1 = alcohol and 0 = other. Frequency of use was coded as 0 = some use or no use in the last month and 1 = daily use.

### Analytic Plan

Chi-squared tests were used to analyze differences in substance use treatment completion across all independent variables. To predict the likelihood of substance use treatment completion, a binary logistic regression was performed, while controlling for the predisposing, enabling, and need factors identified through the Andersen model. To assess multicollinearity among the variables, a test of tolerance and variance inflation factor (VIF) was performed. All tolerance values were less than 1 and all VIF values were less than 10, so the assumptions for a logistic regression were met. For all levels of analysis, alpha level was set at .05.

## Results

### Univariate Results

Descriptive statistics displaying the sample size, percent within sample, and treatment completion percent for each variable are provided in Table 1. A total of 17,942 older adults reported to a substance use treatment program in 2017 and 6,653 reported on all the variables for the study. Of the 6,653, 73.7% completed substance use treatment and 26.3% voluntarily terminated. The majority of the sample were White (65.1%) and male (76.5%). Most of the older adults were divorced, separated, or widowed (44.2%), were not in the labor force (69.7%), and had postsecondary education (41.2%). The most prevalent primary substance of use was alcohol (73.2%) and most of the sample reported using their primary substance only sometimes or not at all in the past month (57.6%).

**Table 1.** Univariate and Bivariate Results

Variable	Values	Sample size (*N*)	% Within sample	% With treatment complete	Pearson χ ^2^	Significance (*p* < .05)
Treatment completion	Treatment completed	4,903	73.7	N/A	N/A	N/A
	Voluntarily terminated	1,750	26.3	N/A	N/A	N/A
Race	Non-Hispanic white	4,330	65.1	76.7	137.688	.000
	Non-Hispanic black	1,599	24.0	62.6		
	Hispanic	724	10.9	80.2		
Gender	Male	5,092	76.5	74.8	12.776	.000
	Female	1,561	23.5	70.2		
Marital status	Married	1,975	29.7	75.2	3.346	.188
	Never married	1,738	26.1	72.8		
	Separated/divorced/widowed	2,940	44.2	73.2		
Employment	Employed	807	12.1	78.6	18.729	.000
	Unemployed	1,206	18.1	76.1		
	Not in labor force	4,640	69.7	72.2		
Education	Has postsecondary education	2,740	41.2	73.2	3.311	.191
	Completed high school	2,565	38.6	74.9		
	Did not complete high school	1,348	20.3	72.5		
Primary substance	Alcohol	4,872	73.2	79.3	293.766	.000
	Other	1,781	26.8	58.4		
Frequency of use	Daily use	2,820	42.4	70.1	33.183	.000
	Some use or no use in the last month	3,833	57.6	76.4		

*Note*: N/A = not applicable.

### Bivariate Results

For treatment completion, significant differences were found across all the groups except marital status and employment. Whites were more likely to complete treatment (76.7%) than Black/African Americans (62.6%). Hispanics had a higher treatment completion rate than Whites and Black/African Americans (80.2%). Males were more likely to complete treatment (74.8%) than females (70.2%).

Married older adults had a higher treatment completion rate (75.2%) than never married (72.8%) and separated/divorced/widowed older adults (73.2%), but this difference was not statistically significant. Older adults who were employed had higher treatment completion (78.6%) than those who were not employed (76.1%) and those who were not in the labor force (69.7%). Older adults who completed at least high school had a higher completion rate (74.9%) than those who had postsecondary education (73.2%) and those that did not complete high school (72.5%), but these differences were not statistically significant.

Those with alcohol as their primary substance had significantly higher completion rates (79.3%) than those who used other substances (58.4%) and finally, those who used their primary substance only sometimes or not at all in the past month had higher completion rates (76.4%) than those who used their primary substance daily (70.1%). The biggest group differences in completion rates were found within primary substance, with a 20.9% difference in completion rates between alcohol users and other substance users. There was also a significant difference in completion rates within race with 17.6% difference between Hispanics and Black/African Americans and a 14.1% difference between Black/African Americans and Whites.

### Multivariate Results

The result of the binary logistic regression is depicted in Table 2. Consistent with the bivariate results, when predisposing, enabling, and need factors were controlled for, race was still a significant predictor of substance use treatment completion. Black/African American older adults were less likely to complete a substance use treatment than Whites (odds ratio [OR] = 0.630), supporting the study hypothesis. This means that White older adults are about 60% more likely to complete a substance use treatment program than Black older adults when the OR is inverted (1/0.630 = 1.6). Hispanics were 26% more likely to complete a substance use treatment program than Whites (OR = 1.263). Males were 29% more likely to complete treatment than females (OR = 1.288).

**Table 2.** Multivariate Results

Variable	Odds ratio	Confidence intervals	Significance *p* < .05
Black/African American compared to Whites	0.630	0.548–0.725	.003
Hispanics compared to Whites	1.263	1.024–1.557	.029
Males compared to females	1.288	1.129–1.471	.000
Never married compared to married	1.102	0.938–1.294	.239
Separated/divorced/widowed compared to married	0.998	0.871–1.143	.974
Unemployed compared to employed	1.013	0.809–1.269	.909
Not in labor force compared to employed	0.799	0.663–0.962	.018
Completed high school compared to postsecondary education	1.225	1.076–1.396	.002
Did not complete high school compared to postsecondary education	1.115	0.948–1.312	.188
Primary substance is alcohol compared to other	2.407	2.120–2.733	.000
Primary substance is used sometimes or not at all in the past month compared to daily use	1.224	1.092–1.372	.045

Marital status was not significant; there was no notable difference in the completion rates between those who were married and those who were either never married or separated/divorced/widowed. There was also no statistically significant difference between those who were employed and those who were unemployed, but there was a significant difference between those who were employed and those who were not in the labor force. Those who were not in the labor force were less likely to complete substance use treatment than those who were employed (OR = 0.799). This means that employed older adults are 25% more likely to complete substance use treatment than older adults who are not in the labor force (1/0.799). Older adults who completed only high school were more likely to complete treatment than older adults who had postsecondary education (OR = 1.225). There was no statistically significant difference in the treatment completion rates between older adults who never finished high school compared to older adults who had postsecondary education.

Older adults with alcohol as their primary substance were over twice as likely to complete a substance use treatment program than those with other substances (OR = 2.407) and older adults who used their primary substance sometimes or not at all in the past month were 22% more likely to complete treatment than those who used it daily (OR = 1.224).

## Discussion

Substance use as an issue among older adults is slowly gaining momentum in the substance use literature. With studies showing significant racial disparities in substance use treatment completion rates in the general population (Chhatre & Jayadevappa, 2018; Gurnack & Johnson, 2002; Mennis & Stahler, 2016; Vasilenko et al., 2017), the increasing trend of older adults reporting to a substance use treatment program warranted an assessment of these disparities across the life span (Chhatre et al., 2017; Gfroerer et al., 2003; Mattson et al., 2017).

For substance use treatment completion rates between Hispanics and Whites, the results of this study show that Hispanic older adults have a higher completion rate both in the bivariate and multivariate analyses compared to White older adults. Previous studies have found varying treatment completion rates for Hispanics compared to Whites, with some studies identifying minor differences (Niv et al., 2009; Perron et al., 2009) and others finding great disparities (Saloner et al., 2014; Stahler & Mennis, 2018). For example, Mennis and Stahler observed that Hispanics had a lower treatment completion rate than Whites, but it was only an 8% difference and was not found to be statistically significant (Mennis & Stahler, 2016). Furthermore, the same study found that the substance of use moderated treatment completion rates, with Hispanics having a higher completion rate than Whites for alcohol but a much lower completion rate for heroin (Mennis & Stahler, 2016). Considering that the majority of the sample used alcohol as their primary substance, the higher completion rates among Hispanics compared to Whites might be a reflection of the moderating effect from type of substance used.

Another possible factor that was not accounted for in the study is the role of social support and the family bond. While marital status was accounted for, the influence of a social network in terms of nonspousal support was not considered. Hispanics have been found to have a cultural preference for family closeness and extended family living arrangements, which results a greater number of people within their social network (Campos et al., 2014). Loyalty to the extended family is extremely important through a concept known as *familismo*, where decisions about health treatments is a group process and a steady network of family members are available to provide advice and encouragement (Calzada et al., 2013). While being married is often viewed as protective factor, children and extended family members may also be a great source of support. With the unique cultural trait of *familismo*, Hispanics may have a higher treatment completion rate because of a greater support system.

In terms of substance use treatment completion rates between Blacks and Whites, Blacks were found to be 34% less likely to complete treatment than Whites. This means that for every 10 White older adults that complete substance use treatment, only six Black older adults complete treatment. These results show a great disparity in substance use treatment completion that have been observed in other similar studies assessing the general population, with Black/African American older adults having a significantly lower treatment completion rates than Whites (Arndt et al., 2011; Jacobson et al., 2007; Mennis & Stahler, 2016).

While the treatment completion rates between Hispanics and Whites is inconsistent across multiple studies, there is a strong consensus when it comes to the disparity between Blacks and Whites, which is often attributed to differences in socioeconomic status (Saloner & Cook, 2013). However, this study shows that even when predisposing, enabling, and need factors are accounted for, there are significant differences in substance use treatment completion between Black and White older adults. This suggests the need to look at race theories to better explain underlying causes of this prevalent disparity.

One possible approach is to use the critical race theory framework to examine the historical, cultural, and systemic context of the health care experiences of Black/African Americans (Ortiz & Jani, 2010; Pulliam, 2017). From the critical race theory perspective, Black/African American older adults may have less substance use treatment completion for several reasons. Dovidio and colleagues (2008), for example, have found that experiences of racial bias and aversive racism have resulted in a distrust of the health care system among Black older adults. On a systemic level, this can be seen in terms of the geographic placement of health care facilities, the cost of services, the availability of insurance coverage, and the quality of the service relative to its location (Dovidio et al., 2008).

Another barrier for Black/African older adults might be stigma, both internalized and public. Negative public view towards mental health prevents older adults from seeking treatment, despite having the intention to do so (Conner et al., 2010a) and African American older adults may feel the need to adhere to culturally acceptable coping strategies, of which seeking external intervention from a professional is not often positively viewed (Conner et al., 2010b). African American older adults from traditional backgrounds may also feel a responsibility to be an “exemplary elder” and have feelings of shame associated with any deviation from that expectation (Lichtenstein, 2008) as in the case of substance use disorders.

In practice, this calls for a culturally sensitive approach to treatment retention for Black/African American older adults in substance use treatment programs, and on a grand political scheme, it calls for a total overhaul of systems that have negatively influenced Black/African American perception of the health care system. On a public level, the need to destigmatize substance use in general is critical, and the expectations of older adults to be model citizens without fault must be diminished. Older adults, like many younger adults, have risk factors that make them susceptible to substance use dependency including chronic pain, social isolation, depression, suicidal ideation, and despair (Arndt et al., 2011; Assari et al., 2019; Cleary et al., 2017; Thandi & Browne, 2019; Millar et al., 2017), and Black/African American adults in particular have “double jeopardy” as a result of their membership in two vulnerable groups (Ferraro, 1987).

### Limitations

Although this study utilized the most comprehensive and recent account of substance use program outcomes available from the SAMHSA, only state-funded treatment program outcomes are reported (SAMHSA, 2019b), which may exclude older adults in private treatment programs, private correctional facilities, and programs like Alcoholics Anonymous. This study only evaluated older adults aged 65 and older, which excludes a significant amount of people in older adulthood, and the inclusion of adults aged 50–65, for example, might yield different results. Similarly, only 37% of the total sample was included in the final sample of the study, which may result in attrition bias. Those who were not included in the study, for example, may have unique characteristics relevant to the study that may have yielded different results. The nature of “voluntary” treatment completion is also ambiguous as older adults may voluntary terminate treatment for reasons such as the death of a loved one, health reasons, family emergencies, and other factors that do not delineate a willingness to quit substance use treatment. Furthermore, the study did not employ a full range of possible predictors that have in the past been found to be relevant when examining racial disparities in substance use treatment, such as the role of social network, type of treatment program, insurance/payment methods, referral source, and geographic residence.

## Conclusion

Substance use among older adults has been largely overlooked due to the inconspicuous nature of this vulnerable population. Several factors associated with aging put older adults at risk of substance use dependency, and this risk is increased for Black/African American older adults who are members of two vulnerable groups. This study contributed to the substance use literature by showing the prevalence of racial disparities in substance use treatment completion across the life span, with results that support previous studies involving younger adults. Despite the consideration of predisposing, enabling, and need factors that are typically proposed as causes of racial disparities in health care utilization, the disparity between Black and White older adults remained. This calls for a consideration of cultural factors in practice, an overhaul of systems that create distrust of the health care system in Black/African Americans, and a need to destigmatize substance use disorders, especially for older adults who are facing an invisible epidemic.
